# Effects of Ultrasound Treatment on Extraction and Rheological Properties of Polysaccharides from *Auricularia Cornea* var. Li.

**DOI:** 10.3390/molecules24050939

**Published:** 2019-03-07

**Authors:** Yinping Wang, Cuina Wang, Mingruo Guo

**Affiliations:** 1 College of Food Science and Engineering, Jilin University, Changchun, Jilin 130062, China; ypwang0812@163.com (Y.W.); wangcuina@jlu.edu.cn (C.W.); 2 Department of Nutrition and Food Science, College of Agriculture and Life Sciences, University of Vermont, Burlington, VT 05405, USA; 3 Department of Food Science, Northeast Agricultural University, Harbin, Heilongjiang 150030, China

**Keywords:** *Auricularia cornea* var. Li., polysaccharides, ultrasound-assisted extraction, structure, rheological properties

## Abstract

*Auricularia cornea* var. Li. is an edible fungi and polysaccharides in *Auricularia cornea* var. Li. may have bioactive activities. Polysaccharides from *Auricularia cornea* var. Li. (ACP) was extracted using ultrasound-assisted extraction (UAE) method and compared with hot water extraction (HWE) for extraction yield, extraction rate, purity of polysaccharides, microstructure of residues after extraction, preliminary structure and rheological properties of polysaccharides. Optimum conditions for UAE (particle size of 150–200 mesh, water to raw material ratio of 70:1, extraction temperature at 70 °C for 40 min, ultrasonic amplitude of 40%) and HWE (particle size of 150–200 mesh, water to raw material ratio of 60:1, extraction temperature at 90 °C for 3.0 h) were obtained via single-factor experiment. Under optimum conditions, extraction yield of polysaccharides by UAE was 30.99 ± 1.93% which showed no significant difference with that by HWE (30.35 ± 1.67%) (*P* > 0.05). Extraction rate (29.29 ± 1.41%) and purity (88.62 ± 2.80%) of polysaccharides by UAE were higher than those by HWE (extraction rate of 24.95 ± 2.78% and purity of 75.33 ± 6.15%) (*P* < 0.05). Scanning Electron Microscopy (SEM) images of residues by UAE showed more broken cells than those by HWE. Fourier Transform Infrared (FTIR) spectra showed that the dialyzed ACP extracted by HWE and UAE (DACP-HWE and DACP-UAE) had similar characteristic absorption peaks of polysaccharides. Both DACP-HWE and DACP-UAE solutions showed typical shear thinning and temperature-independent behaviors (25–90 °C) and UAE resulted in polysaccharides with remarkably lower viscosity in comparison with HWE. DACP-UAE solutions exhibited more liquid-like state while DACP-HWE solutions solid-like system. Data indicated that ultrasound treatment may be a useful means for extraction of polysaccharides from *Auricularia cornea* var. Li.

## 1. Introduction

Edible fungi contain various bioactive components including polysaccharides, polyphenol, protein and vitamins [1]. Especially, edible fungi polysaccharides have attracted attention due to its potentially medicinal values, such as anti-oxidative [2], antitumor [3,4], anti-proliferation [5] and hypoglycemic activities [6]. In recent years, novel techniques including enzyme-assisted extraction [7], microwave-assisted extraction [8,9] and ultrasound-assisted extraction [10] have been applied for extraction of polysaccharides from edible fungi. Among all the methods, enzyme-assisted extraction has advantages being environmentally friendly and high efficient [11], however, the enzyme activity could be easily influenced by several factors [12]. Microwave-assisted extraction could enhance extraction efficiency with less time consumption [8]. It may have the disadvantage of inhomogeneous heating [13]. Ultrasound-assisted extraction (UAE) is a popular means due to quick and high efficiency [14,15]. It could improve the yield of polysaccharides by disrupting the cell walls, reducing the particle size and enhancing contact between solvents and targeted compounds due to the acoustic cavitation effect [16]. 

Studies on rheological properties of polysaccharides may provide useful information for their application as potential thickeners and stabilizers in food industry [17,18]. Bao et al. [19] reported that water-soluble polysaccharides from *Auricularia auricular-judae* exhibited desirable functional properties including high viscosity with shear-thinning behavior and forming thermal stable gels ability, which were useful for application in food industry. Ma et al. [17] investigated effects of different concentration, temperature and pH on rheological properties of polysaccharides from *Dioscorea opposite Thunb*, which contributed to the function and applications of natural thickeners in food industry. 

Previous studies showed that rheological properties of polysaccharides could be affected by ultrasound treatment due to reduction of the molecular weight of polysaccharides [20]. Li et al. [21] reported that high intensity ultrasound reduced average molecular weight, particle size of konjac glucomannan aggregates without significant structural destruction and changed the rheological properties including the decrease of storage modulus (G’) and loss modulus (G’’) and the increase in phase angle. Zhong et al. [20] investigated molecular weight degradation of schizophyllan under ultrasonic treatment and showed that the reduction of viscosity and elastic behavior of polysaccharides may be related to its lower molecular weight after ultrasonic treatment. Seshadri et al. [22] studied the effect of high intensity ultrasound processing on rheological properties of high-methoxyl pectin dispersions and showed that cavitation effect from ultrasound induced the reduction in average molecular weight and changed the flow behavior of pectin, which had important implications for future applications of food hydrocolloids in food industry.

Edible fungi are regarded as a valuable source of nutritional and bioactive ingredients and have been used in dietary and medicinal applications for thousands of years [1,23]. *Auricularia cornea* var. Li. (“Yu Mu Er” in Chinese), a white-body edible fungi, is a variant of *Auricularia cornea* species [24]. It is rich in bioactive compounds such as dietary fiber and polysaccharides and has been cultivated and commercialized in China [25].

The objectives of this study were to extract polysaccharides from *Auricularia cornea* var. Li. using UAE and compared with hot water extraction (HWE) in terms of extraction yield, extraction rate, purity of polysaccharides, microstructure of residues after extraction. The optimum conditions for both extraction methods were obtained by single-factor experiment. The preliminary structure and rheological properties of polysaccharides extracted by HWE and UAE under the optimum conditions were also investigated. 

## 2. Results and Discussion

### 2.1. Effects of Single-Factor by Ultrasound-assisted Extraction on Extraction Yield and Extraction Rate of Polysaccharides from Auricularia cornea var. Li.

**Figure 1:**
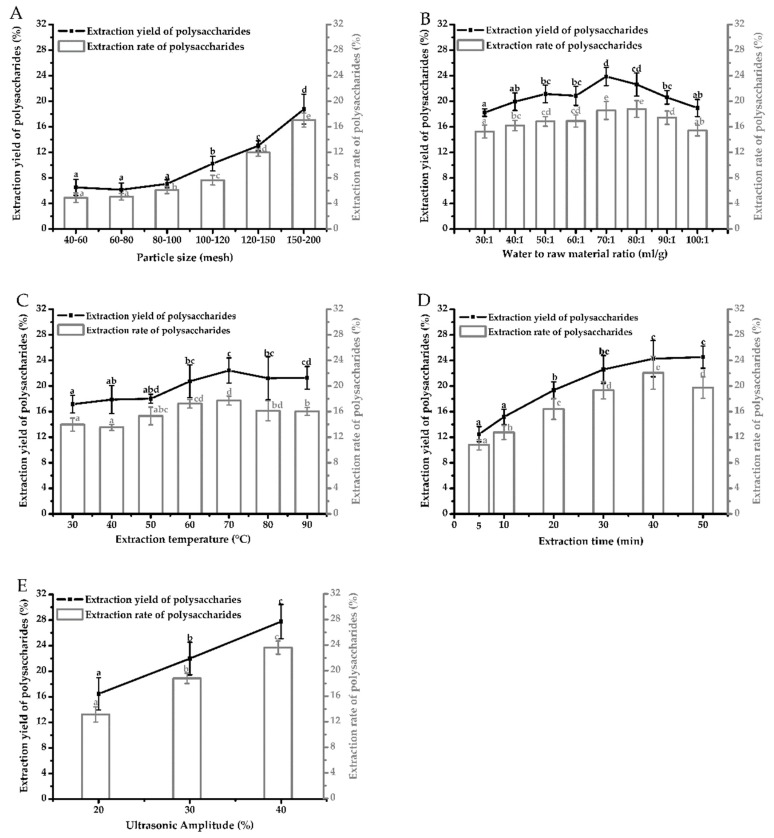
Effects of single-factor (**A**) particle size (**B**) water to raw material ratio (**C**) extraction temperature (**D**) extraction time (**E**) ultrasonic amplitude by ultrasound-assisted extraction (UAE) on extraction yield and extraction rate of polysaccharides from *Auricularia cornea* var. Li. Values with different letters are significantly different (*P* < 0.05) for extraction yield and extraction rate of polysaccharides.

Effects of 5 factors (particle size, water to raw material ratio, extraction temperature, extraction time, ultrasonic amplitude) by UAE on extraction yield and extraction rate of polysaccharides from *Auricularia cornea* var. Li. were evaluated and shown in Figure 1A–E.

Particle size was an important factor which can affect the extraction yield and extraction rate of polysaccharides [26]. Effects of particle size (40–60, 60–80, 80–100, 100–120, 120–150, 150–200 mesh) on extraction yield and extraction rate were evaluated at constant conditions of water to raw material ratio of 60:1 (mL/g), extraction temperature of 60 °C, extraction time of 20 min, ultrasonic amplitude of 30%. As shown in Figure 1A, no significant changes were found in extraction yield and extraction rate when particle size decreased from 40–60 to 60–80 mesh (*P* > 0.05). Notably, both extraction yield and extraction rate of polysaccharides increased significantly as particle size decreased from 80–100 to 150–200 mesh (*P* < 0.05). This indicated that more polysaccharides were transported from interior of particles to the solvent. Smaller particle sizes in certain range would result in high extraction efficiency due to a smaller resistance and shorter path to the polysaccharides diffusion between the raw material and solution [26]. However, some studies also reported that promotion of extraction yield may be impeded due to mass transfer resistance started to become larger when particle size continually reduced [27]. Powders at particle size of 150–200 mesh showed the highest extraction yield (18.77 ± 2.34%) and extraction rate (17.07 ± 1.10%) among all samples. Thus, 150–200 mesh was selected as the optimum particle size.

Water to raw material ratio in UAE was set at 30:1, 40:1, 50:1, 60:1, 70:1, 80:1, 90:1, 100:1 (mL/g) and other extraction conditions were as following: particle size of 150–200 mesh, extraction temperature of 60 °C, extraction time of 20 min, ultrasonic amplitude of 30%. As shown in Figure 1B, extraction yield and extraction rate of polysaccharides increased when water to raw material ratio increased from 30:1 to 70:1 (*P* < 0.05), then decreased rapidly with further increase from 80:1 to 100:1 (*P* < 0.05). Samples at ratio of 70:1 and 80:1 showed larger extraction yield and extraction rate (*P* < 0.05). Increasing the water to material ratio may improve the diffusion rate between the interior of raw material and the exterior solvent and enhanced the dissolution of polysaccharides [28,29]. However, too much solvent may prolong the distance of diffusion which may cause further loss of polysaccharides [29,30]. Water to raw material ratio of 70:1 (mL/g) with relatively high extraction yield (23.88 ± 1.43%) and extraction rate (18.58 ± 1.41%) was chosen as the optimum ratio to avoid solvents wasting.

Extraction temperature was set at 30, 40, 50, 60, 70, 80, 90 °C with particle size of 150–200 mesh, water to raw material ratio of 60:1 (mL/g), extraction time of 20 min, ultrasonic amplitude of 30%. As shown in Figure 1C, extraction yield and extraction rate of polysaccharides had no significant increase when extraction temperature was increased from 30 to 50 °C (*P* > 0.05). When extraction temperature increased from 50 to 70 °C, it had a positive effect on extraction yield and extraction rate (*P* < 0.05). No significant changes were observed on extraction yield and extraction rate slightly decreased with increasing temperature from 70 to 90 °C (*P* < 0.05). High temperature could increase the dissolution of polysaccharides from the material. Too high temperature may lead to the degradation of polysaccharides [31,32]. Extraction temperature of 70 °C was selected as the optimum extraction temperature, in which extraction yield and extraction rate were the highest (22.44 ± 1.96% and 17.73 ± 0.70 %).

Samples with particle size of 150–200 mesh were dissolved at ratio of 60:1 (mL/g) and treated at ultrasonic amplitude of 30% and extraction temperature of 60 °C for various times (5, 10, 20, 30, 40, 50 min). As shown in Figure 1D, extraction time displayed positive effects on extraction yield and extraction rate when increased from 5 to 40 min (*P* < 0.05). No significant change was observed in extraction yield (*P* > 0.05) and significant decrease in extraction rate was observed when extraction time was further prolonged to 50 min. Ultrasound treatment could enhance the release and diffusion of polysaccharides easily into the solvents but long-time exposure to ultrasound treatment may induce their breakage and degradation [33,34,35]. Thus, extraction time of 40 min was selected as optimum extraction time which had higher extraction yield (24.29 ± 2.83%) and the highest extraction rate (22.09 ± 2.58%).

Ultrasonic amplitude was one of the key factors and it was set at 20, 30, 40%. Other extraction conditions were as following: particle size of 150–200 mesh, water to raw material ratio of 60:1 (mL/g), extraction temperature of 60 °C, extraction time of 20 min. As shown in Figure 1E, extraction yield and extraction rate increased significantly (*P* < 0.05) with increasing ultrasonic amplitude. Increase in ultrasonic amplitude could strengthen the effect of cavitations and promote cell wall fragmentation, which increased the diffusion and dissolution of polysaccharides [29]. Ultrasonic amplitude of 40% was chosen as the optimum level for further study where the highest extraction yield (27.76 ± 2.68%) (*P* < 0.05) and extraction rate (23.59 ± 1.03%) (*P* < 0.05) were observed.

### 2.2. Effects of Single-Factor by Hot Water Extraction on Extraction Yield and Extraction Rate of Polysaccharides from Auricularia cornea var. Li.

**Figure 2:**
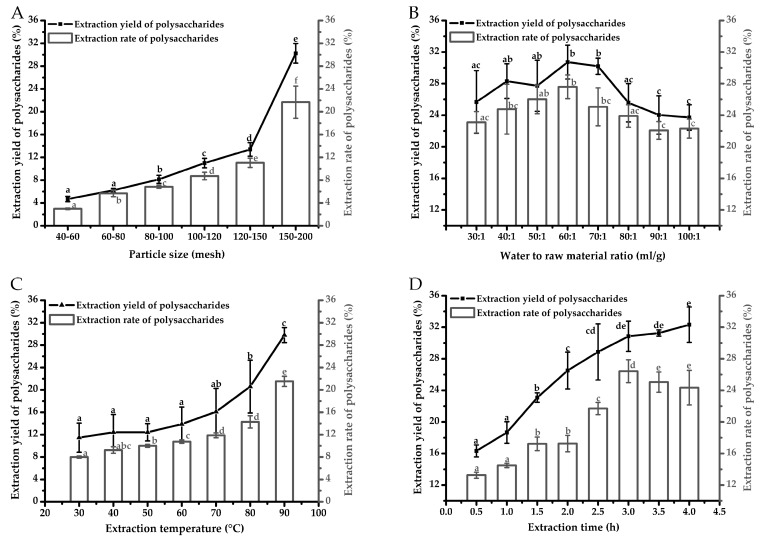
Effects of single-factor (**A**) particle size, (**B**) water to raw material ratio, (**C**) extraction temperature, (**D**) extraction time by hot water extraction on extraction yield and extraction rate of polysaccharides from *Auricularia cornea* var. Li. Values with different letters are significantly different (*P* < 0.05) for extraction yield and extraction rate of polysaccharides.

Figure 2A–D showed the effects of 4 factors (particle size, water to raw material ratio, extraction temperature, extraction time) by HWE on extraction yield and extraction rate of ACP. Effects of particle size (40–200 mesh) on extraction yield and extraction rate in HWE were shown in Figure 2A. Extraction yield had no significant change (*P* > 0.05) when particle size decreased from 40 to 80 mesh. However, extraction yield increased significantly from 8.15 ± 0.71% to 30.24 ± 1.72% as particle size decreased from 80–100 to 150–200 mesh (*P* < 0.05). Extraction rate of polysaccharides increased significantly from 2.96 ± 0.14% to 21.68 ± 2.83% as particle size decreased from 40–60 mesh to 150–200 mesh (*P* < 0.05). Thus, optimum particle size was 150–200 mesh which showed the highest extraction yield (30.24 ± 1.72%) and extraction rate (21.68 ± 2.83%) (*P* < 0.05). As shown in Figure 2B, extraction yield of polysaccharides had no significant change when water to raw material ratio ranged from 30:1 to 50:1 (*P* > 0.05). There was a slight increase from 50:1 to 60:1. No significant difference was observed between samples with ratio of 60:1 and 70:1 (*P* > 0.05). However, extraction yield decreased rapidly from 30.20 ± 1.03% to 23.72 ± 1.61% when water to raw material ratio ranged from 70:1 to 100:1 (*P* < 0.05) and had no significant change with ratio increasing from 90:1 to 100:1. No significant decrease in extraction rate was observed as ratio increased from 70:1 to 100:1. Thus, water to raw material ratio of 60:1 had the highest extraction yield (30.73 ± 2.15%) and extraction rate (27.60 ± 1.51%) and was selected as the optimal ratio. Effects of different extraction temperature (30-90 °C) on extraction yield and extraction rate were shown in Figure 2C. Extraction yield had no significant increase when extraction temperature increased from 30 to 60 °C (*P* > 0.05). Extraction yield and extraction rate increased rapidly within the increase of extraction temperature from 60 to 90 °C (*P* < 0.05). Therefore, the optimum extraction temperature in HWE was 90 °C. Effects of extraction time on extraction yield and extraction rate were studied with different extraction time of 0.5, 1.0, 1.5, 2.0, 2.5, 3.0, 3.5, 4.0 h (Figure 2D). Extraction yield increased from 16.33 ± 0.76% to 30.86 ± 1.92% with the extending of extraction time from 0.5 to 3.0 h (*P* < 0.05) and had no significant increase (*P* > 0.05) when extraction time exceeded from 3.0 to 4.0 h. Extraction rate increased significantly with extraction time increasing from 2.5 to 3.0 h (*P* < 0.05). Higher extraction yield (30.86 ± 1.92%) and the highest extraction rate (26.43 ± 1.45%) were obtained when extraction time increased to 3.0 h. Thus, optimum extraction time in HWE was 3.0 h. 

### 2.3. Extraction Yield, Extraction Rate, Purity of Polysaccharides by Ultrasound-assisted Extraction and Hot Water extraction

Optimized extraction conditions of UAE and HWE were summarized in Table 1. The optimum conditions of UAE were shown as follows: particle size of 150–200 mesh, water to raw material ratio of 70:1 (mL/g), extraction temperature at 70 °C for 40 min, ultrasonic amplitude of 40%. The optimum conditions of HWE were shown as follows: particle size of 150–200 mesh, water to raw material ratio of 60:1 (mL/g), extraction temperature at 90 °C for 3.0 h. As shown in Table 1, extraction yield of polysaccharides by UAE (30.99 ± 1.93%) with shorter extraction time had no significant difference with that of HWE (30.35 ± 1.67%) (*P* > 0.05). Extraction rate (29.29 ± 1.41%) and purity (88.62 ± 2.80%) of polysaccharides by UAE were all higher than those (extraction rate of 24.95 ± 2.78% and purity of 75.33 ± 6.15%) by HWE (*P* < 0.05). 

### 2.4. Microstructure

**Figure 3:**
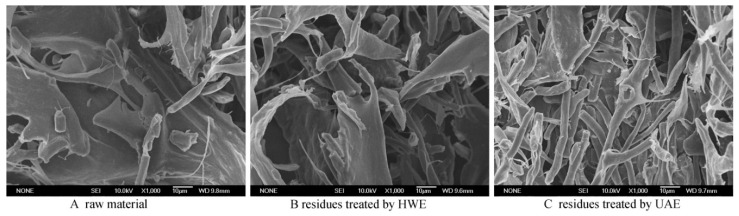
Scanning Electron Microscopy (SEM) images of *Auricularia cornea* var. Li. (**A**) raw material, (**B**) residues treated by HWE, (**C**) residues treated by UAE.

Microstructure of *Auricularia cornea* var. Li. and residues treated by UAE and HWE were observed and the images were shown in Figure 3A–C. Ultrasound-assisted extraction and hot water extraction methods resulted in different physical changes of residues. Compared with untreated material, microstructure of residues treated by HWE had no significant difference, while residues treated by UAE showed significant change. Heated solvent in HWE diffused slowly through the cell walls, dissolved and carried away polysaccharides slowly, which had weak effect on cell tissues of material [16]. By contrast, ultrasound treatment could produce acoustic cavitation in liquids which could induce physical changes [36] and the cell tissues of residues treated by UAE (Figure 3C) were explosively disrupted which was benefit for the diffusion of polysaccharides [7]. Scanning Electron Microscopy (SEM) images of residues extracted after ultrasound treatment showed that more broken cells with rod-like shape may be responsible for the high extraction rate of polysaccharides.

### 2.5. Fourier Transform Infrared Spectra 

**Figure 4:**
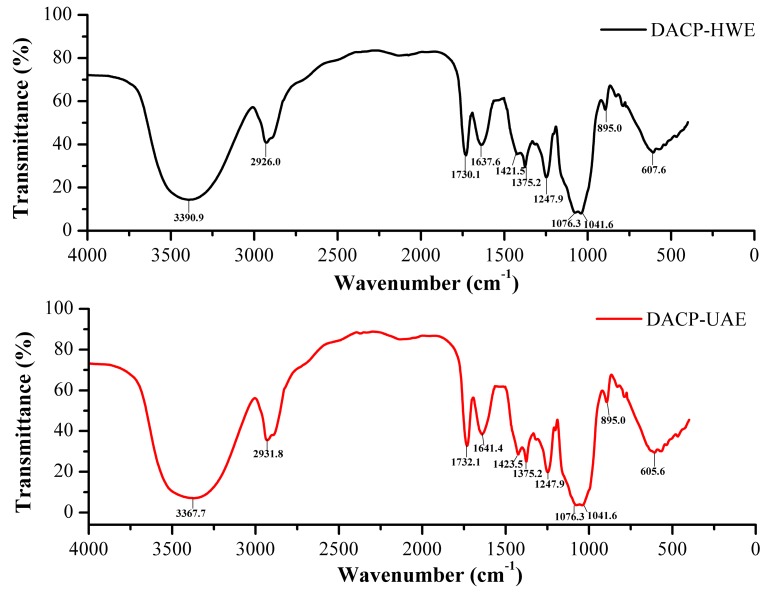
Fourier Transform Infrared (FTIR) spectra of dialyzed ACP (DACP)-HWE and DACP-UAE.

Figure 4 showed the Fourier Transform Infrared (FTIR) spectra of dialyzed ACP (DACP)-HWE and DACP-UAE, which had virtually identical characteristic absorption peaks. Two characteristic absorptions of polysaccharides, the strong and broad peaks at around 3390.9 cm^−1^ (DACP-HWE) and 3367.7 cm^−1^ (DACP-UAE) for O–H stretching vibrations and the weak peaks at around 2926.0 cm^−1^ (DACP-HWE) and 2931.8 cm^−1^ (DACP-UAE) for C-H stretching vibrations were observed, respectively [37,38]. The bands at approximately 1730.1 cm^−1^ (DACP-HWE), 1732.1 cm^−1^ (DACP-UAE), 1637.6 cm^−1^ (DACP-HWE), 1641.4 cm^−1^ (DACP-UAE) were attributed to stretching vibration of C=O [28,39]. The weak peaks observed at 1421.5 cm^−1^ (DACP-HWE), 1423.5 cm^−1^ (DACP-UAE) and 1375.2 cm^−1^ indicated the stretching vibration of C–H [40]. Peaks observed at 1247.9 cm^−1^, 1076.3 cm^−1^ and 1041.6 cm^−1^ may be assigned to the contribution of C–O–C symmetric stretching vibration [13,41,42]. Absorption peaks at 895.0 cm^−1^ suggested the presence of β-configurations in the molecular structure of DACP-HWE and DACP-UAE [42,43]. The FTIR spectra showed all typical absorption peaks associated with polysaccharides which confirmed the identity of DACP-HWE and DACP-UAE as polysaccharides.

### 2.6. Rheological Properties 

#### 2.6.1. Steady-Shear Flow Property

**Figure 5:**
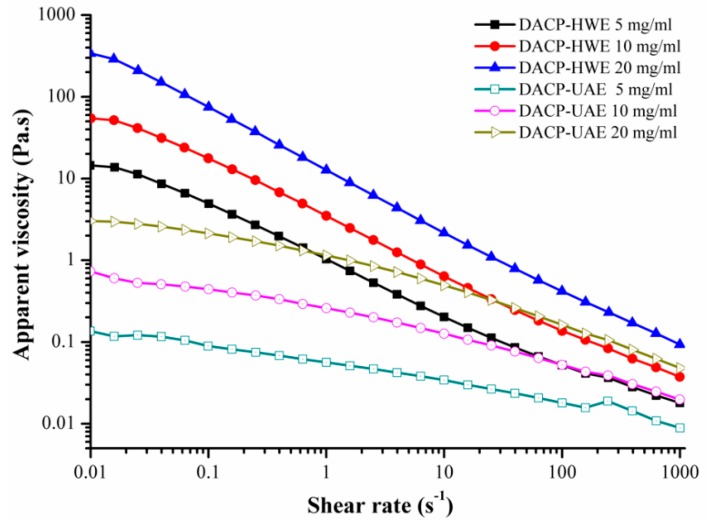
Steady shear flow curves of DACP-HWE and DACP-UAE solutions with different concentrations (5, 10, 20 mg/mL) as a function of shear rate.

The flow properties of DACP-HWE and DACP-UAE solutions with different concentrations (5, 10, 20 mg/mL) were investigated using steady shear flow sweeps in the shear rate range of 0.01–1000 s^−1^ (Figure 5). The apparent viscosity of DACP-HWE and DACP-UAE solutions with different concentrations decreased with increased shear rate and all showed typical shear-thinning behaviors. Similar results were observed for polysaccharides of *Auricularia auricular-judae* and other polysaccharides [19,21,44]. The flow curves of DACP-UAE were similar with those of DACP-HWE that apparent viscosity increased significantly with increase of the concentrations in the shear rate range of 0.01–1000 s^−1^. As shown in Figure 5, when concentration of DACP-HWE and DACP-UAE solutions were increased from 5 to 20 mg/mL, apparent viscosity at shear rate of 0.1 s^−1^ were increased from 4.92 to 74.76 Pa.s and from 0.09 to 2.14 Pa.s, respectively. Increased entanglement and aggregation of molecule chains at higher concentrations may be responsible for higher viscosity [45]. However, apparent viscosity of DACP-UAE was significantly lower than that of DACP-HWE with same concentration at the same shear rate. Similar results were observed for schizophyllan [20]. Decrease in apparent viscosity of DACP after ultrasound treatment could be attributed to the reduction in molecular weight [21,46,47,48]. 

**Figure 6:**
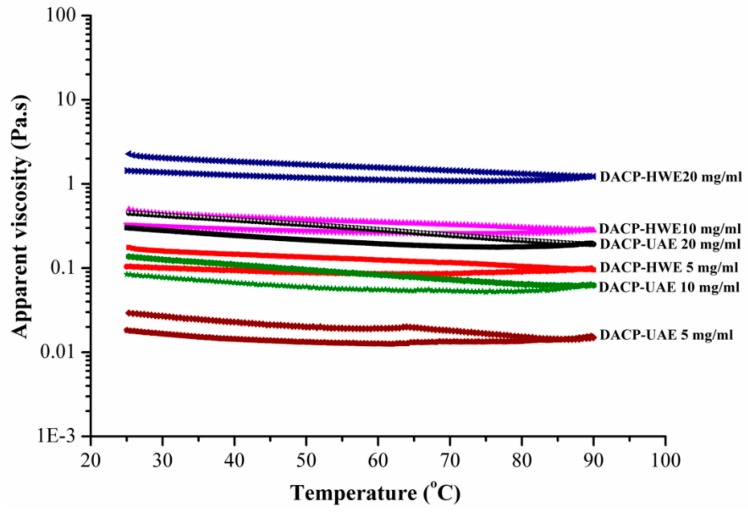
Steady shear flow curves of DACP-HWE and DACP-UAE solutions with different concentrations (5, 10, 20 mg/mL) as a function of temperature (25–90 °C).

All samples were determined for apparent viscosity by flow temperature ramp at shear rate of 10 s^−1^ (Figure 6). The results were consistent with the steady flow curves and apparent viscosity increased with increase of concentration. Apparent viscosity of DACP-HWE solutions was higher than that of DACP-UAE at same concentration. Both DACP-HWE and DACP-UAE solutions showed temperature-independent behaviors, indicating the non-sensitivity of temperature at 25 to 90 °C. Similar results were reported for polysaccharides of *Auricularia auricular-judae* [19]. 

#### 2.6.2. Dynamic Viscoelastic Property

**Figure 7:**
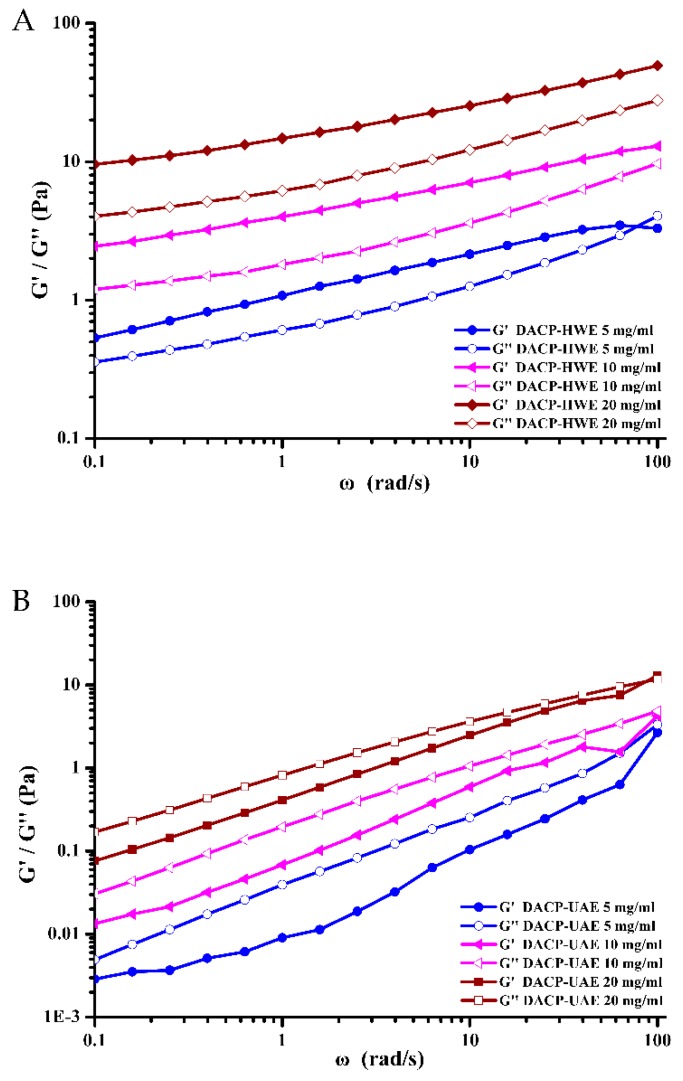
Dynamic storage modulus (G’) and loss modulus (G’’) with oscillatory frequency sweeps of (**A**) DACP-HWE solutions with different concentrations (5, 10, 20 mg/mL) and (**B**) DACP-UAE solutions with different concentrations (5, 10, 20 mg/mL).

Frequency-dependent behaviors of DACP-HWE and DACP-UAE solutions with different concentrations were examined within the linear viscoelastic range (LVR) at frequency range of 0.1–100 rad/s (Figure 7A,B). Storage modulus (G’) and loss modulus (G’’) of DACP-HWE and DACP-UAE solutions were observed as a function of frequency (ω) and all increased with increasing frequency. For DACP-HWE solutions at the concentrations of 10 and 20 mg/mL, G’ were higher than G’’ within the frequency range (0.1–100 rad/s) and both had a tendency to approach each other at a high frequency (no crossover point occurred), which exhibited the typical gel-like behaviors. Similar results have been reported in previous studies [19,20]. The DACP-HWE solution at 5 mg/mL exhibited a transition from a gel-like to a fluid-like structure in which G’ was higher than G’’ in low frequency range followed by the domination of G’’ with a crossover point at a high frequency. The crossover point occurred at ω = 77.61 rad /s and G’ = G’’= 3.40 Pa. For DACP-UAE solutions of 5 mg/mL and 10 mg/mL, G’’ were higher than G’ in the frequency range (0.1–100 rad/s) and both had a tendency to approach each other at a high frequency which exhibited the typical fluid-like behaviors [49]. As the increase of concentration, DACP-UAE solution at 20 mg/mL exhibited the transition from a fluid-like to a gel-like structure in which G’’ was higher than G’ in low frequency range followed by the domination of G’ with a crossover point at a high frequency. The crossover point occurred at ω = 86.89 rad /s and G’ = G’’= 11.01 Pa. Ultrasound treatment decreased the elastic behavior and increased the viscous system of ACP [20].

## 3. Materials and Methods

### 3.1. Materials

*Auricularia cornea* var. Li. (“Yu Mu Er”) was purchased from Jilin Agricultural University, China. The standard monosaccharide (D-glucose) was purchased from Sigma (St. Louis, MO, USA). Dialysis membranes (molecular weight cut off, 8 kDa) were purchased from Solarbio (Beijing, China). All other chemicals were of reagent grade and purchased from Beijing Chemical Works (Beijing, China). Deionized water used in this study was made by Millipore Milli-Q™ water purification system (Millipore Corp., Bedford, MA, USA).

### 3.2. Extraction of Polysaccharides from Auricularia cornea var. Li.

**Figure 8:**
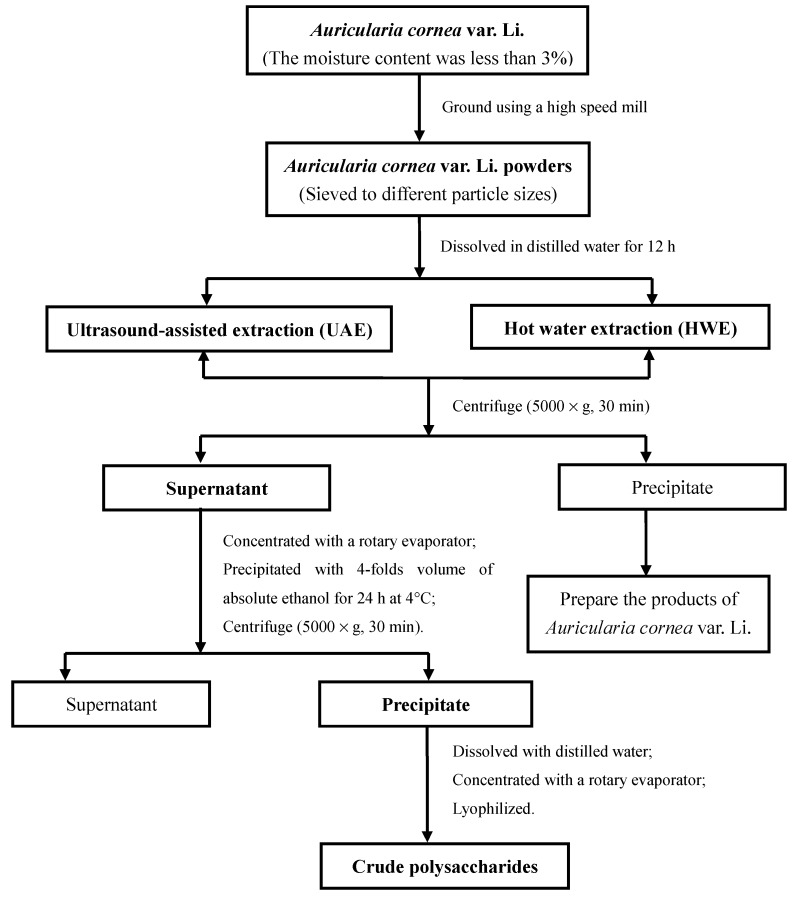
Flow chart and experimental design for the extraction process of polysaccharides from *Auricularia cornea* var. Li.

*Auricularia cornea* var. Li. was dried in an oven at 45 °C until its moisture content was less than 3%. Then the dried sample was ground using a high speed mill (FW100, Tianjin, China) and sieved into powders with a series of standard sieves (Shaoxing, Zhejiang, China) with various particle sizes (40, 60, 80, 100, 120, 150, 200 mesh size screen). The sieves were consistent with National Standard (GB/T 6003.1-2012). Crude polysaccharides from *Auricularia cornea* var. Li. (ACP) were extracted using UAE and HWE and the detailed extraction process was shown in Figure 8. 

#### 3.2.1. Ultrasound-assisted Extraction of Polysaccharides from *Auricularia cornea* var. Li.

Ultrasound-assisted Extraction (UAE) was performed in a 800 W, 20 kHz ultrasonic processor (VCX800, vibra cell, Sonics, Newtown, CT, USA) with a 13 mm high grade titanium alloy probe (amplitude, 114 μm) threaded to a 3 mm tapered microtip to sonicate the *Auricularia cornea* var. Li. powder dispersions in a 200-ml jacketed glass beaker [50]. *Auricularia cornea* var. Li. powder (2.0 g) under designated size was mixed with distilled water (water to raw material ratio of 30:1–100:1, mL/g) in conical flasks and stirred using magnetic stirrers (IKA Ared, Pedrollo, Italy) for sufficient dissolution and then treated with ultrasonic probe (20 kHz) at certain extraction temperature and ultrasonic amplitude for required time. The extraction temperature was preset with thermostatic temperature control and the pulse duration was on-time 10 s and off-time 5 s. Effects of particle size (40–200 mesh), water to raw material ratio (30:1–100:1, mL/g), extraction temperature (30–90 °C), extraction time (5–50 min) and ultrasonic amplitude (20–40%) were studied by a single-factor experiment. After extraction, the extract was centrifuged at 5000× *g* for 30 min using a centrifuge (Avanti^®^ J–E, Beckman Coulter, Calif., USA). The supernatant was concentrated with a rotary evaporator (N-1100, EYELA, Tokyo, Japan) and precipitated with 4-folds volume of absolute ethanol for 24 h at 4 °C. The polysaccharides precipitate was obtained by centrifugation at 5000× *g* for 30 min and then dissolved in distilled water. Polysaccharides solutions were removed for residual ethanol and concentrated with the rotary evaporator (N-1100, EYELA, Tokyo, Japan); and lyophilized (Alpha 1–2 LDplus, CHRIST, Osterode am Harz, Germany) to obtain crude polysaccharides.

#### 3.2.2. Hot Water Extraction of Polysaccharides from *Auricularia cornea* var. Li. 

*Auricularia cornea* var. Li. powder (2.0 g) was mixed with distilled water (water to raw material ratio of 30:1–100:1, mL/g) in conical flasks and stirred using magnetic stirrers for sufficient dissolution and then extracted under water bath condition with designated particle size (40–200 mesh), extraction temperature (30–90 °C) and extraction time (0.5–4 h). The following processes for obtaining the crude polysaccharides were similar to UAE. 

#### 3.2.3. Calculation of Extraction Yield, Extraction Rate, Purity

Polysaccharides content was determined by phenol-sulfuric acid method using D-glucose as standard reference [51]. Extraction yield (%), extraction rate (%) and purity (%) were calculated using following equations according to previous studies [7,26,40]:

(1) $$\mathrm{Extraction}\;\mathrm{yield}\;\left(\%\right)=W_{1}/W_{0}\times 100$$

(2) $$\mathrm{Extraction}\;\mathrm{rate}\;\left(\%\right)=\;W_{2}/W_{0}\times 100$$

(3) $$\mathrm{Purity}\;\left(\%\right)\;=\;W_{2}/W_{1}\times 100$$

where *W*_0_ is the weight of dried *Auricularia cornea* var. Li. powder (g), *W*_1_ is the weight of dried crude polysaccharides (g), *W*_2_ is the content of polysaccharides (g).

### 3.3. Scanning Electron Microscopy Analysis

To investigate the effect of HWE and UAE on the microstructure of materials, Scanning Electron Microscopy (SEM) was used to reveal the shape and surface characteristics of samples after extraction [16]. Dried samples (raw material, residues treated by UAE and HWE) of *Auricularia cornea* var. Li. were fixed on a specimen holder with the help of doubled-sided adhesive tapes and then sputtered with gold powder. Microstructure was obtained using a Scanning Electron Microscope (JMS-6700F, JEOL Co., Tokyo, Japan) at a 10 kV acceleration voltage.

### 3.4. Fourier Transform Infrared Spectroscopy Analysis

Under the optimum conditions, crude polysaccharides from *Auricularia cornea* var. Li. (ACP) extracted by HWE and UAE were dialyzed using dialysis membranes (molecular weight cut off, 8 kDa) against distilled water for 24 h with the stirring of magnetic stirrer, then lyophilized. The dialyzed ACP extracted by HWE and UAE was abbreviated as DACP-HWE and DACP-UAE. 

Fourier Transform Infrared (FTIR) spectra of DACP-HWE and DACP-UAE were analyzed using an IR Prestige-21 Spectrometer (Shimadzu Co, Ltd, Kyoto, Japan) in the range of 400–4000 cm^−1^. Approximately 2 mg samples (DACP-HWE and DACP-UAE) were mixed with 200 mg dried KBr and then pressed into pellets for FTIR measurement [52].

### 3.5. Rheological Property Measurements

#### 3.5.1. Sample Preparation 

The dialyzed samples (DACP-HWE and DACP-UAE) were dispersed in deionized water at different concentrations (5, 10, 20 mg/mL) and stirred for sufficient dissolution at room temperature. Dispersions were kept at 4 °C overnight to enable biopolymer hydration and release of bubbles [53]. 

#### 3.5.2. Steady-Shear Flow Property

Rheological measurements of DACP-HWE and DACP-UAE solutions were carried out using a rheometer (DHR-1, TA Instrument, New Castle, DE, USA) with a aluminum parallel plate geometry (diameter = 60 mm, plate spacing = 1 mm) [54]. Samples were placed on the plate of rheometer and excessive samples were wiped off with a clean paper towel. 

Flow sweep analysis was conducted on DACP-HWE and DACP-UAE solutions with different concentrations at shear rate ranging from 0.01 to 1000 s^−1^ at 25 °C. Apparent viscosity was recorded as a function of shear rate. 

Flow temperature sweep analysis at the shear rate of 10 s^−1^ was performed on DACP-HWE and DACP-UAE solutions with different concentrations from initial temperature of 25 °C to 90 °C with a rising rate of 3 °C/min and then cooled to 25 °C at the same rate. 

#### 3.5.3. Dynamic Viscoelastic Property

For the dynamic oscillatory frequency sweep analysis, variations in storage modulus (G’) and loss modulus (G’’) of DACP-HWE and DACP-UAE solutions as a function of frequency (ω) (0.1–100 rad/s) were tested at 25 °C. Before tests, the linear viscoelastic range (LVR) of DACP-HWE and DACP-UAE solutions with different concentrations were established using strain sweeps at a constant frequency of 1 Hz (Figure S1). Frequency sweeps were performed with a strain of 5% which was within the linear viscoelastic range for all samples.

### 3.6. Statistical Analysis

All experiments were performed in triplicate. Statistical analyses were carried out using statistical program IBM SPSS Statistics Version 21.0. (SPSS Inc. Chicago, IL, USA). Comparisons among data of different groups were performed with One-way ANOVA, where LSD method and Dunnet’T3 were used on the basis of the homogeneity test. Results were presented as mean ± standard deviation (SD) and considered significantly different when *P* < 0.05 at 95% level of confidence. Figures were drawn using Origin 8.0 (Origin lab Corp., Northampton, MA, USA).

## 4. Conclusions 

Ultrasound-assisted extraction was an effective method for extracting polysaccharides from *Auricularia cornea* via. Li. It showed high extraction rate and the extracted polysaccharides have high purity, relatively low apparent viscosity and liquid-like behavior. The preliminary structure of polysaccharides was not changed by UAE compared with conventional hot water extraction method.

## Figures and Tables

**Table 1:** Comparison of extraction yield, extraction rate, purity of polysaccharides from *Auricularia cornea* var. Li. (ACP) extracted by hot water extraction (HWE) or ultrasound assisted extraction (UAE).

Methods	HWE	UAE
Particle size (mesh)	150–200	150–200
Water to raw material ratio (mL/g)	60:1	70:1
Extraction time	3.0 h	40 min
Extraction temperature (°C)	90	70
Ultrasonic amplitude (%)	/	40
Extraction yield of polysaccharides (%)	30.35 ± 1.67 ^a^	30.99 ± 1.93 ^a^
Extraction rate of polysaccharides (%)	24.95 ± 2.78 ^a^	29.29 ± 1.41 ^b^
Purity of polysaccharides (%)	75.33 ± 6.15 ^a^	88.62 ± 2.80 ^b^

Note: Different letters in row indicate significant differences at *P* < 0.05.

## Supplementary Materials

The following are available online, Figure S1: The Linear Viscoelastic Range (LVR) of DACP-HWE and DACP-UAE solutions with different concentrations (5, 10, 20 mg/mL)
