# Supplementary material for: Effects of Ultrasound Treatment on Extraction and Rheological Properties of Polysaccharides from Auricularia Cornea var. Li
Source: Molecules. 2019 Mar 7;24(5):939. doi: 10.3390/molecules24050939 (PMC6429225; doi:10.3390/molecules24050939)
Supplement: Supplementary file 1 [file molecules-24-00939-s001.pdf]

molecules

Supplementary Materials

Effects of Ultrasound Treatment on Extraction and Rheological Properties of Polysaccharides from *Auricularia cornea* var. Li.

Yinping Wang [1], Cuina Wang [1] and Mingruo Guo [2,3,*]

[1] College of Food Science and Engineering, Jilin University, Changchun, Jilin, 130062, China; ypwang0812@163.com (Y.W.); wangcuina@jlu.edu.cn (C.W.)
[2] Department of Nutrition and Food Science, College of Agriculture and Life Sciences, University of Vermont, Burlington, Vermont 05405, USA
[3] Department of Food Science, Northeast Agricultural University, Harbin, Heilongjiang 150030, China
* Correspondence: mguo@uvm.edu; Tel.: +1-(802) 656-8168; Fax: +1-802-656-0001

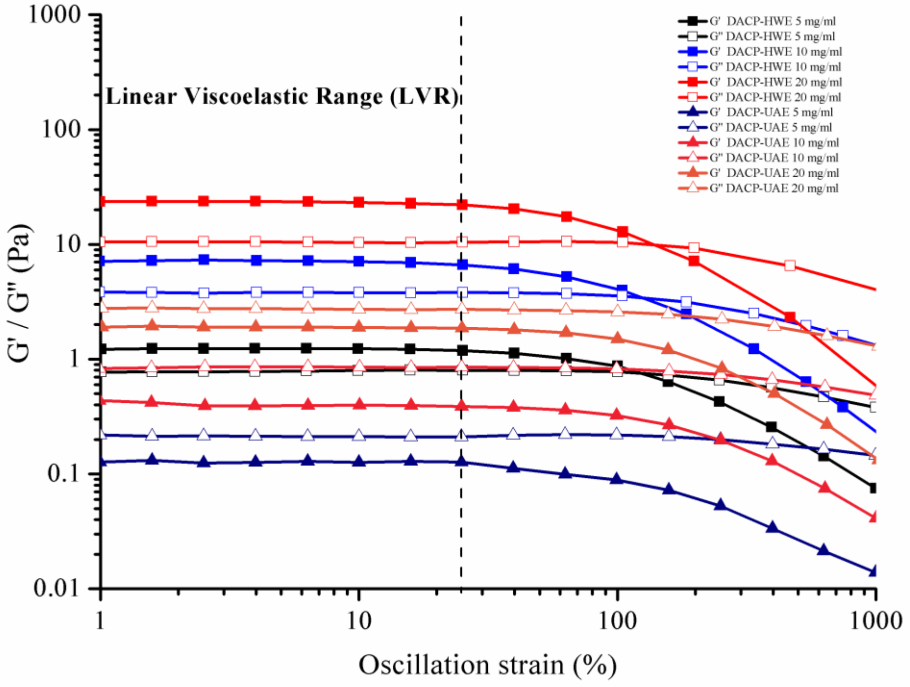

Figure S1. The Linear Vicoelastic Range (LVR) of DACP-HWE and DACP-UAE solutions with different concentrations (5, 10, 20 mg/ml).
